# The Importance of a High Grade of Physical Health in the Effort to Cure Develop, or Reform the Dependent and Delinquent Classes Cared for in Public Institutions*Read at a meeting of the American Social Science Association, held at Saratoga, September, 1897.

**Published:** 1898-01

**Authors:** William P. Spratling

**Affiliations:** Sonyea, N. Y., Medical Superintendent Craig Colony for Epileptics


ATLANTA

Medical and Surgical Journal.

VOL. XIV. JANUARY, 1898. No. 11.

L. B. GRANDY, M.D., DUNBAR ROY, M.D., EDITORS. M. B. HUTCHINS, M.D., BUSINESS MANAGER.

### ORIGINAL COMMUNICATIONS.

### THE IMPORTANCE OF A HIGH GRADE OF PHYSICAL HEALTH IN THE EFFORT TO CURE, DEVELOP, OR REFORM THE DEPENDENT AND DELINQUENT CLASSES CARED FOR IN PUBLIC INSTITUTIONS.*

By WILLIAM P. SPRATLING, M.D., SONYEA, N. Y.,

Medical Superintendent Craig Colony for Epileptics.

I am honored by the invitation of the chairman of the Department of Health of this Association to take part in discussing the importance of a high grade of physical health in the effort to cure, develop, or reform the dependent and delinquent classes cared for in public institutions.

It is a most timely topic; timely for the reason that the day of simple, indiscriminate custodial care for such classes is rapidly becoming a forgotten practice, and for the added reason that the greater economy lies in curing or reforming such classes as against giving them custodial care to be perpetuated through the entire lifetime of the dependent individual.

*Read at a meeting of the American Social Science Association, held at Saratoga, September, 1897.

Statistics recently came under my observation of a French family of dependents, where the initial fault lay in the mother four generations back, and whose progeny was so numerous and defective through the four succeeding generations that their care imposed upon the French government an outlay of one and a quarter million dollars.

But the money value of such care cannot be compared to the distress and suffering that followed this family and its immediate connections through four generations. The public charities of this country, and particularly of the great commonwealth of New York, have grown to fairly astounding proportions, and some of the millions of dollars that are being spent annually do not go into the channel, the current of which flows to cure, development and reform, but leads into the stagnant waters of simple custodial care, pregnant with the inaction of routine practices and fruitless of beneficent results.

In this State and elsewhere there are notable exceptions to this, but they only serve to accentuate the too widely prevalent policies that varying circumstances force the use of in caring for the dependent and delinquent classes. Sometimes it is politics; at others a lack of money; or again it may be laid at the door of a cause just as disastrous as either of the above, namely, inaction.

My province in this discussion has to do with the epileptic, and whatever may be said hereafter in this paper on the subject of sound health, how to attain it, and its value to the dependent classes, will be in its application to the epileptic. And my remarks will be under two heads: the one theoretical, or what it is possible to do; the other practical, or what has been done.

These will be prefaced by a brief description of the disease from which the epileptic suffers. "A sound mind in a sound body" is so old an adage and so commonly used that we do not stop to consider the wealth of its meaning. Yet the sum total of the entire complex problem we are called on to consider lies wholly within its meaning. If the entire human organism is sound in all its parts; if that soundness extends to and includes the nucleus of every cell and protoplasmic mass, and to the outermost ramifications of every nerve fiber, there can be but one condition of the body as a whole, and that will be one of perfect physiologic health and soundness. But such a condition would be an anomaly, it would be so rare.

Epilepsy was described by Hippocrates thirty centuries ago, and from that day to this its treatment has been as persistent as ambition could make it and as varied as the resources of the materia medica, combined with the art and skill of the physician and surgeon, could devise.

Epilepsy is generally described as being a paroxysmal affection, characterized by convulsive movements and followed by loss of consciousness. The latter may or may not be true, since we know that certain types of seizures do occur without loss of consciousness. The attacks may be foreshadowed by a warning, or they may come without warning. The convulsive movements vary in degree from the most delicate twitching of the finer muscles about the mouth, to convulsive shocks so universal and violent as to throw the patient forcibly to the ground. They may occur as often as every three minutes or less apart, or they may be separated by intervals of months, or even years.

The mental and physical deterioration that epileptics are so prone to suffer from differs widely in degree and depend originally upon the physical and mental stamina of the individual, and later, on the frequency and severity of the seizures.

It is largely a disease of childhood and early life, since seventy per cent. of all cases begin under the age of twenty years; and this is an important fact to remember when we come to study its treatment.

The causes that produce it are legion, but the etiological factor of greatest importance is heredity; thirty-five and thirty-seven per cent. of all cases in males and females, respectively, being ascribed to that cause. After heredity comes a long list of causes as numerous as are the varieties of the disease itself, but which may be stated in the concrete to include the specific and non-specific diseases of a general nature, and their sequelæ; the various forms of auto-intoxicants; the numerous excesses; shock; fright, and causes removable through surgical procedures.

Referring again to the factors in heredity, the so-called stigmata of degeneration must be mentioned. They include asymmetry of the face and cranium, protruding teeth, malformed ears, and various deformities of the hard palate.

To what extent these influences are responsible for the production of epilepsy I do not know, but it is my belief that if they mean anything at all, it must be that they stand as part evidence of the fact that the structural organization of the individual, as compared with the normal standard, is incomplete; and since it is incomplete, in this respect we have some warrant for assuming that some omission has been made from the integral structure of the central nervous system of the individual at fault.

Out of 145 cases studied at the Craig Colony, including both sexes and all ages from eight to seventy years, and among whom, individually, the disease had existed for from one to forty years, 131 of them presented evidences of the stigmata of degeneration. And in the treatment of the epileptic, in trying to give him a higher grade of physical health as a preface to curing his disease, we must constantly recognize the presence of congenital deficiencies and not vainly strive to supply what nature originally omitted. Among those, then, of the public dependents who demand a high grade of physical health as a prerequisite to the cure of their disease, the epileptic stands in the foremost rank.

Not only does it too often happen that congenital deficiencies handicap the possibility of his recovery, but the very nature of his malady is such as to rob him of whatever mental and physical stamina he may possess. And this will seem all the more strange when we realize that convulsions are far nearer normal than the stormy nature of their presence would lead us to believe. But we will not wonder so much at this if we go just a step further and briefly study the mechanism of the fit itself. We assume that all manifestations witnessed during a convulsion appear as the result of a sudden liberation of nerve force somewhere in the brain. It is not often that a seizure occurs without involving, sooner or later, the motor cells of the cortex of the brain; and as a rule these cells constitute the seat of the discharging lesion more frequently than any other. We go a little further and are led to believe that the discharge in the motor cells of the cortex takes place as the result of defective inhibitory action on the part of the sensory cells underlying the cortical layers. Just why these sensory cells partially or wholly lose control periodically over the action of the motor cells above has not been explained, but like the stigmata of degeneration, the forces of inhibition may have suffered from lack of completion in the original creation.

The epileptic, therefore, is not only often congenitally weak, but is peculiarly liable to acquire a very generally marked condition of feeble health. All this being true, the need of the highest possible degree of physical health that can be attained through proper and continuous exercise and means of development is all the more forcibly demanded, and in seeking to bring this about we should not for a moment forget the one fact important above all others in developmental training in the restoration of health, and that is: that the very exercise that strengthens and develops the muscles of the arms and legs, or indeed any other part of the body, will just as surely strengthen and develop the area or centers in the brain that control these outlying parts.

Theoretically and without any violence to the teachings of physiology, we should be able, under proper given conditions, to take a young and healthy body and train it up to the highest possible standard of physical health.

We need only to remember that with the young, nervous system anything is possible, and that, roughly speaking, the plasticity of the nerve cells declines in proportion to their age. It is this fact that makes early education so important.

The system of developmental training, the training that carries with it, coequally, all parts and functions of the body, and that brings with it perfect physical health, is so elaborate in its construction that it can be only touched on in this paper. Its first principle lies in the recognition of coequal development. The rest is a matter of method and detail. Coequal development means that all of the functions and special sense faculties of the body shall share, in proportionate degree, in the processes of education and development. Muscular development should go hand in hand and keep equal pace with the growth and expansion of the brain; and the brain should not be forced to work under forced draught while the muscular system is permitted to atrophy through habitual inaction. Inaction means atrophy, and atrophy is closely allied to disease.

Many vocations call for the habitual use of a given set of muscles, or for brain work along sharply circumscribed lines, and the individual called upon to do such work will in time be overdeveloped, as far as health is concerned, in one direction, and suffer from lack of development in another. The tendencies of the times are toward specialisms, and the laws of progress demand not only the survival of the fittest but the development of specialized faculties. But it is not healthy and the wreckage of health that follows the practice is becoming greater all the while.

When we come to the employment of means for the promotion of health for the epileptic, we do not need to use any system different from that we would use to strengthen and develop a normal individual. But with the epileptic we must begin earlier in point of age, work harder, and in the end be satisfied to have accomplished less.

And by no means should we fall into the disastrous error of taking a fatalistic view of the case; for, in spite of all his deficiences, his abnormally low standard of physical health, his ingrained inertia, his too frequently perverted moral sense, I know of no class of sick or defective dependents for whom more can be done than for the epileptic.

Under proper measures, almost miraculous changes can be made in his condition, and that too through influence devoted solely to building him up physically. As to the best methods of doing this I can only briefly indicate in this paper, but I hope brevity will not contract the expansive horizon of the lesson it teaches.

Recognizing to the fullest extent the great value of a high grade of physical health for the development and cure of the epileptic, I declare to this Association that it is my belief that the solution of the problem will be found to be largely solved when we come to recognize to the fullest extent the great value of industrial education for this class.

Hidden in the mazes of its great diversity of forms, all of which contain the possibilities of physiological and moral regeneration, lie, almost wholly to this time unrevealed, potential influences destined to work untold good, under proper seeking and cultivation by the dependent and delinquent classes.

The principle of labor was given prominence above all others when the now famous colony for epileptics at Bielefeld, Germany, was founded thirty years ago; and it has been kept foremost on the list of remedial agents to this day. We recognize its value at the Craig Colony for Epileptics, because we have fully tested it and in no instance found it wanting. As yet our system of industrial education is incomplete; indeed, far from perfect, but it is growing and it is our aim to extend it for both sexes, as far as it is possible to go.

We have found that labor, properly and systematically performed, is a moral and regenerative agent of untold value. And why? Simply because it brings about a physiological and healthy growth of the entire organism.

We believe in labor; the kind that quickens the pulse and brightens the eye; that sends currents of pure blood sweeping throughout the entire vascular system; the kind that brings normal fatigue and induces sweet sleep; the labor that has a place in the world of economics; and the kind that eventually conquers all things, even the perverted tendencies of a degenerate ancestry. The value of systematic labor for the epileptic is but little appreciated. It has curative powers not lacking for proof.

I would not be understood as saying that work will cure all cases of epilepsy; for nothing could be more absurd than such a statement. But I make the assertion, and can demonstrate it to the entire satisfaction of the most skeptical, that the healthy physiological activity necessary in systematic labor acts as a normal avenue of escape for the accumulated energy that would otherwise expend itself in a convulsion.

I could cite a score of cases in support of this assertion; cases in which the connection between fits and no work and no fits and work is so patent as to leave possible but one deduction.

V. S., male; age twenty-six; was admitted to the colony February 5, 1896. He had been an epileptic for eighteen years. Twice previously he had been discharged from State hospitals as incurable, hopeless. For five years prior to his admission to the colony he averaged from three to five seizures a day. During his first month at the colony he had 110 seizures; during the second 98; during the third 3 seizures; during the fourth no seizure; during the fifth 1 seizure, and that was brought on through excitement caused by his going away from the colony over night, and for fifteen months now he has not had a seizure. He has learned the printer's trade at the colony, and is now doing most of the printing required by the colony. When admitted he was emaciated and weak, and had to be supported to his meals by two persons. We got him out doors for a little exercise daily, and he had a generous diet of proper food. When potato-planting commenced, and just as soon as he could get about, he was put in the field with a bag of potatoes over his shoulder and required to work an increasing length of time each day. He had medicines, of course, but they had no more effect on him than they had during the previous years of his disease.

There was only one reason why he improved under the new form of treatment; he was made to acquire, through labor, better physical health, and when that came the vulnerable points for attack on the part of his epilepsy were destroyed and the attacks ceased.

While this single case is reported, I might report fully a score where the value of labor has been none the less marked.

For the cure, development and reform of the dependent and delinquent classes, I believe we must first elevate the standard of physical health of these classes, and this I believe can be best done through the medium of the wide diversity of industrial training that seems so full of promise, both from the standpoint of economy and from that yet more valuable, the physical and moral regeneration of an ever-increasing class.

---

RECENTLY a woman in New York city, who went into a store to buy a well-known medicine, was persuaded to take something "just as good." She took it and died from the effects of it. A suit for damages is pending.—*Ex.*

